# Phenotypic and functional heterogeneity of peripheral γδ T cells in pulmonary TB and HIV patients in Addis Ababa, Ethiopia

**DOI:** 10.1186/s12879-018-3361-9

**Published:** 2018-09-15

**Authors:** Mikias Negash, Aster Tsegaye, Liya Wassie, Rawleigh Howe

**Affiliations:** 1 College of Health Sciences, Department of Medical Laboratory Science, Addis Ababa University, Addis Ababa, Ethiopia; 2 Armauer Hansen Research Institute, Addis Ababa, Ethiopia

**Keywords:** Peripheral γδ T cells, Vδ1, Vδ2 T cell subsets, Pulmonary TB, HIV

## Abstract

**Background:**

Previous studies reported HIV infection alters the distribution and function of γδ T cells and their subsets. γδ T phenotypes in healthy and diseased individuals has received little attention in Ethiopia. We conducted this study to analyze the distribution of γδ T cells, the subsets and levels of expression of activation (CD38), exhaustion or anergy (CD95, PD1), adhesion (N-CAM/CD56 and CD103), among HIV and TB infected patients.

**Method:**

The distributions of total γδ T cells, Vδ1 and Vδ2 T cells subsets were analyzed in clinical samples collected from asymptomatic HIV, pulmonary TB patients and apparently healthy controls. Multicolor flow cytometry and IFN-γ ELISA were used to assess surface markers and functional responses of Vδ2 T cells to isopentenyl pyrophosphate stimulation, respectively.

**Result:**

A total of 52 study participants were enrolled in this study, 22 HIV + TB-, 10 HIV-TB+ and 20 healthy controls. No significant differences were observed in the distribution of total γδ T cells and in the proportion of Vδ1 subsets in all study groups, though slightly higher proportions were observed in HIV + TB- patients for the latter, of borderline statistical significance (*p* = 0.07). However, the proportion of Vδ2 T cells, as well as the IFN-γ response to IPP stimulation, was significantly reduced in HIV + TB- patients compared to healthy controls (*p* < 0.002). Expression of the activation marker CD38 (*p* < 0.001) and adhesion marker CD103 (αEβ7) were significantly higher in the Vδ1 T cell subset among both HIV + TB- (*p* = 0.013) and HIV-TB+ (*p* = 0.006) patients compared to healthy controls. Similarly, exhaustion markers, CD95 and PD1, were significantly higher in these two T cell subsets among both HIV + TB- and HIV-TB+ patients (*p* < 0.01). Interestingly, we also observed an increased proportion of effector memory (CD45RA-CD27-) and effector cytotoxic (CD45RA + CD27-) Vδ2 T cell subsets in HIV negative pulmonary TB patients.

**Conclusion:**

In sum, HIV infection was associated with an increase in Vδ1 and a decrease in the function and frequencies of Vδ2 T cells. Moreover, increased effector Vδ2 T cells were observed among HIV negative pulmonary TB patients suggesting a potential role of these T cells in the host response to TB.

## Background

γδ T cells are minor subset, 1–5%, among circulating T cells, but are present in abundance within mucosal lymphoid tissue, therein comprising as much as 50% of T cells. The two main subsets are Vδ1 and Vδ2 T cells [1]. The Vδ1^+^ γδ T cells are mainly situated at mucosal sites and respond to non-classical major histocompatibility complex molecules such as MICA and/or MICB expressed on stressed cells [2]. The Vγ9Vδ2 T cell subset on the other hand are dominant in the peripheral circulation and respond to phospholigands (non-peptide molecules) derived from potentially diverse microbes, including mycobacteria [3]. A role for Vγ9Vδ2 cells in anti-microbial immune defense is suggested by an increase in the activation and proliferation of these T-cells in response to intracellular pathogens, such as *Mycobacterium tuberculosis* [3, 4].

In vitro, Vγ9Vδ2 T cells have been shown to possess diverse immune activities. Vγ9Vδ2 T cells showed activation and expansion upon culture with HIV-infected lymphocytes [5], and this activation has been associated with direct cytotoxic activity mediated by the Fas/Fas-L interactions and by cytotoxic granules. Indirect anti-microbial activities of Vγ9Vδ2 T lymphocytes include roles in Th1 T-cell polarization and dendritic cell (DC) maturation [6].

Prior studies have demonstrated a reduced expression of CD28 and an increased expression of CD38 and HLA- DR on γδ T cell populations of HIV patients, findings also seen with conventional α/β T cells, suggesting the possibility that all circulating T lymphocyte share similar phenotypic abnormalities in HIV infected patients [7]. Similarly, HIV infected patients were shown to have an increased level of Vδ1 [8, 9] and a decreased level of Vδ2 cells [9–11]. Although there has been no clear conclusion, the increased Vδ1 T cell subset may have been due to redistribution from mucosal areas as a result of changes in cytokine levels [6] rather than HIV antigen driven expansion [8, 12]. Notably, the reduction in the proportion and function of the Vδ2 subset was marked in patients with advanced AIDS stage, and this partially recovered after active antiretroviral therapy (ART) [10, 11].

There are conflicting data on the frequency or functional response of total γδ and Vδ2 T cells in pulmonary TB patients. Some studies have reported an increase in frequency and function of γδ T cells and Vδ2 subsets in TB patients [13]*,* while others reported a decrease in Vδ2 subsets frequency and function following whole blood stimulation with phosphoantigen [14]; still others reported no differences when compared to healthy controls [15, 16]. Given the inconsistency of previous reports, as well as lack of information on these T cells profile in the Ethiopian setting, the aim of this study was to assess the distribution of γδ T cells and their subsets Moreover, the expression of activation, adhesion and exhaustion markers was measured to further reveal the possible role these T cells play in such diseases.

## Methods

### Study setting and population

This cross-sectional study, conducted between February 2015 to June 2016, involved a total of 52 participants recruited from a tertiary health facility, the anti-retroviral treatment (ART) follow up clinic of the All African Leprosy and Rehabilitation Training Center (ALERT Hospital), Addis Ababa and two additional medium sized health centers, Arada Health Center and Teklehaimanot Health Center in Addis Ababa.

Twenty two of the participants were HIV positive TB negative patients and 10 were HIV negative pulmonary TB patients. The HIV positive TB negative cases were all asymptomatic, free of any opportunistic infections and were on HAART for at least 2 years before inclusion into the study, whereas the HIV negative TB patients were treatment-naïve pulmonary TB patients, presenting with clinical TB signs and symptoms and confirmed with acid fast bacilli (AFB) smear microscopy. Additionally, 20 age and sex matched apparently healthy individuals were recruited as controls from Voluntary Counseling Testing centers (VCT). Healthy controls with acute illness, chronic disease conditions such as diabetes, hematological malignancies, HIV and lymphomas were excluded from the study. All participants gave written informed consent and the study received ethical approval from institutional review committee at the Department of Medical Laboratory Science, Addis Ababa University, AHRI/ALERT Ethics Review Committee and the Ethiopian National Research Ethics Review Committee.

### Flowcytometry analysis

Multicolor flowcytometry was used to study the percentage of cells expressing activation, adhesion, exhaustion and memory markers on Vδ1 and Vδ2 T cell subsets in HIV + TB- and HIV-TB+ patients, and healthy controls. Typically, 8 ml of peripheral venous blood was collected from eligible participants in heparin tubes and dispensed and lysed in polystyrene Florescence Activated Cell Sorter (FACS) tubes using red blood cell (RBC) lysis buffer (0.15 M Ammonium chloride). Following lysis, leukocytes were washed with phosphate buffered saline (PBS), and resuspended in FACS buffer (1 mg/ml bovine serum albumin, 1 mM EDTA in PBS). Cells were subsequently stained for cell surface markers using fluorochrome conjugated monoclonal antibodies, namely Vδ2-PE, TCR γδ-PE, CD56-APC, CD8-APC-Cy7, CD38-V450, CD3-V500, CD95-V450, CD27-V500, CD45RA-PE-Cy7, all from Beckton Dickinson (BD Biosciences, Belgium), Vδ1-FITC (ThermoFisher, South Africa) and PD1-APC, CD103-PE-Cy7 (eBioscience,USA). Cells were incubated for 20 min at 4 °C, washed with FACS buffer and fixed with 2% paraformaldehyde. Data was acquired on a FACSCanto II flow cytometer using FACSDiva software (BD Biosciences). Analysis of lymphocyte sub-population frequencies and the relative expression of the different markers was performed with FlowJo Version 9.9.4 Software (TreeStar Inc., USA) after gating on CD3^+^ T lymphocyte subsets expressing markers for Vδ1^+^ and Vδ2^+^ (see Fig. 2).

### IPP-specific IFN-γ response whole blood stimulation assay

For this experiment, 100 μl of heparinized whole blood was dispensed into 96 well plates (BIORAD, USA) in duplicate and was stimulated with or without 50 μM isopentenyl pyrophosphate (IPP) (Sigma Aldrich, USA) in RPMI 1640 medium supplemented with 10% Fetal Calf Serum, 1% L -glutamine, penicillin, and streptomycin. After 24 h incubation in 5% CO_2_ incubator at 37 °C, supernatants were harvested from each well for detection of IPP-specific IFN-γ production using an Enzyme Linked Immunosorbent Assay (ELISA) (BIORAD, USA).

The data obtained in the presence of stimuli exhibited Optical Density (OD values above unstimulated controls, but were low and in the non-linear portion of the IFN-γ standard curve. Considering the extrapolation of OD values to IFN-γ concentrations therefore unreliable, and since all culture supernatants were evaluated by ELISA in the same experiment, we opted to simply express data as OD units in the presence of stimulus less that in the absence of stimulus.

### Statistical analysis

The data was entered into excel, cleaned and imported to Statistical Package for Social Sciences (SPSS) Software Version 21.0 (Chicago, IL, USA) for statistical analyses. Frequencies and proportions were used to describe the characteristics of study participants in relation to relevant variables. The non parametric, Mann-Whitney *U* test was used to compare median percentage of cell subsets among different study groups. Graphpad Prism Version 6.0 was used to generate graphs. A *p*-value < 0.05 was considered as statistically significant.

## Results

In this study 52 study subjects participated and were categorized in three groups based on their disease status. The three groups were similar with respect to age and sex. The mean age of the patients was 35.4 and 32.4 for HIV + TB- and HIV-TB+ groups, respectively (Table 1).

**Table 1:** Socio-demographic characteristics of HIV + TB-, HIV-TB+ patients and healthy controls

Study Groups	n	Sex		Age in years
		Male	Female	Mean (SD)
HIV + TB-	22	9	13	35.4 (7.4)
HIV-TB+	10	5	5	32.4 (11.5)
Healthy controls	20	12	8	31.2 (9.4)
Total	52	26	26	

### Distribution of γδ T cells and their subsets in peripheral whole blood

**Fig. 1:**
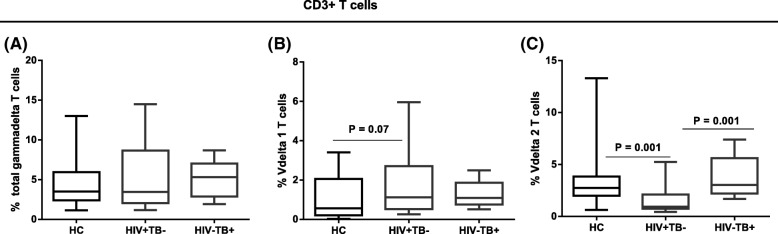
Distribution of total and subsets of gamma-delta T cells. Distribution of total γδ (**a**), Vδ1 (**b**) and Vδ2 T cells (**c**). Data shown are box plots depicting median, intra-quartile range and range of subset frequencies defined from peripheral blood cells of 22 HIV + TB- and 10 HIV-TB+ patients, and 20 healthy controls. *p* values are from the Mann Whitney *U* Test

The median percentage of total γδ T cells among CD3^+^ T lymphocytes was comparable in HIV + TB- subjects and healthy controls with respective medians of 3.7% and 3.5%. HIV-TB+ subjects exhibited a slightly higher median (5.3%) though this value did not reach statistical significance (Fig. 1). Further delineation into Vδ1 and Vδ2 subsets revealed that while Vδ1 percentages were higher in HIV + TB- than in HC subjects (*p* = 0.07, borderline significance), Vδ2 percentages were significantly lower. The percentages of total γδ T cells among HIV-TB+ subjects did not differ from HC (Fig. 1).

### Expression of activation and other markers by Vδ1 and Vδ2 T cells subsets

**Fig. 2:**
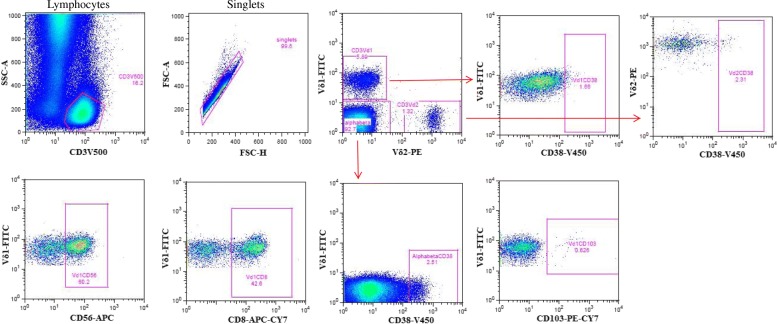
Gating of gamma delta T cells sub-populations and different markers. Gating strategy to define gamma delta T cells, sub-populations thereof, and different surface marker expression from one representative HIV patient. Single cells were gated from CD3+ lymphocytes. Vδ1 and Vδ2 T subsets were defined and these gated subsets were further evaluated for expression of different surface markers. Thresholds to define indistinct markers such as CD38 on γδ T cells were copied from that defined on αβ T cells as depicted in the figure

**Fig. 3:**
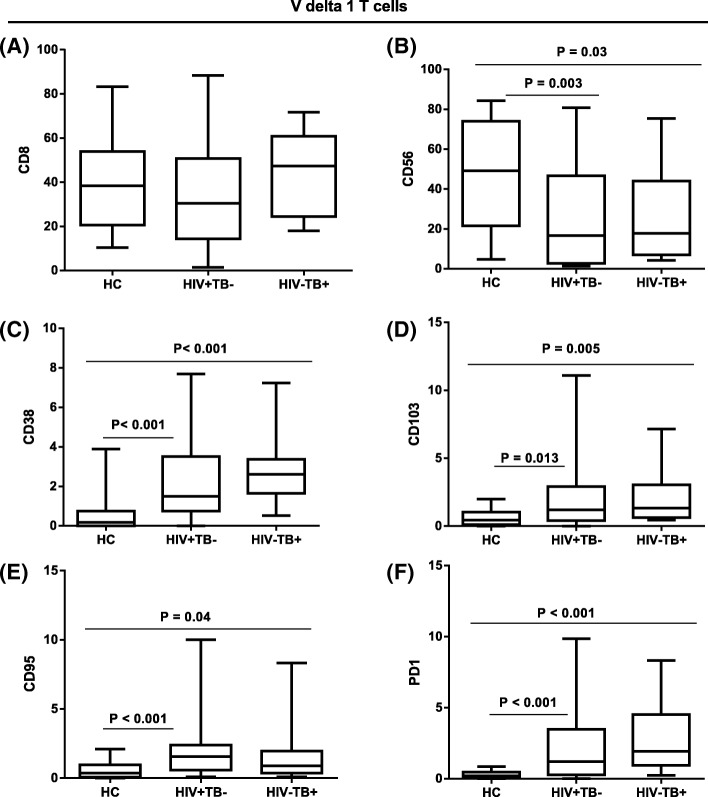
Expression of different biomarkers on Vδ1 subsets. Median percentage of CD8 (**a**), CD56 (**b**), CD38 (**c**), CD103 (**d**), CD95 (**e**), and PD1 (**f**) on Vδ1 T cell subsets from peripheral blood cells of 22 HIV + TB-, 10 HIV-TB+ patients and 20 healthy controls. *p* values are from the Mann Whitney *U* Test

We next defined the expression of a range of other markers related to lineage, adhesion, activation, and exhaustion on the two γδ subsets using a gating strategy as defined in Fig. 2. Our results showed that the median percentage of Vδ1 T cells expressing CD8 did not significantly differ across all three study groups. However, expression of the activation marker CD38 among Vδ1 cells was significantly higher in both patient groups, HIV + TB- and HIV-TB+, compared to healthy controls (*p* < 0.001). Similarly, the frequency of the adhesion marker CD103 in Vδ1 cells was significantly higher in both patient groups, HIV + TB- (*p* = 0.013) and HIV-TB+ (*p* = 0.005), compared to healthy controls. In addition, the expression of exhaustion markers CD95 and PD1 were significantly higher in both patient groups. In contrast, the frequency of CD56 among Vδ1 cells was significantly lower in the patient groups compared to healthy controls (Fig. 3).

**Fig. 4:**
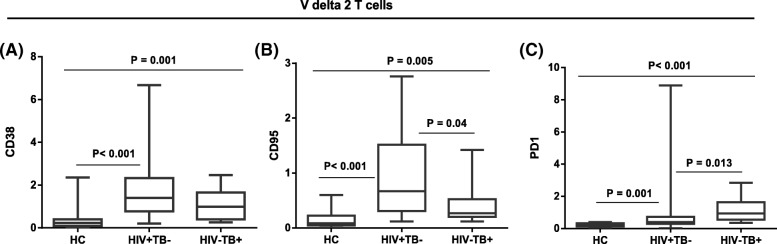
Expression of different biomarkers on Vδ2 subsets. Median percentage expression of CD38 (**a**), CD95 (**b**) and PD1 (**c**) on Vδ2 T cell subsets from peripheral blood cells of 22 HIV + TB-, 10 HIV-TB+ patients and 20 healthy controls. *p* values are from the Mann Whitney *U* Test

Similar to our findings of Vδ1 T cells, the median percentage of CD38 expressing cells within the Vδ2 subset was significantly higher in the two patient groups than healthy controls (*p* < 0.001), as shown in Fig. 4. The frequency of cells positive for the exhaustion markers CD95 and PD1 among Vδ2 T cells were similarly higher in patients compared to healthy controls (*p* = 0.001). HIV negative TB patients had significantly higher proportions of Vδ2 T cells (*p* = 0.002) and PD1 expressing Vδ2 cells (*p* = 0.013) compared to HIV positive TB negative patients.

### Expression of differentiation markers by Vδ2 T cells subsets

**Fig. 5:**
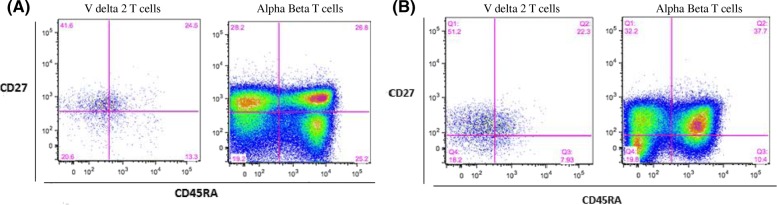
Gating of memory markers on conventional T cells and gamma delta T cells. Frequency of Vδ2 cells stained with CD27 and CD45RA mAbs. Peripheral blood cells from 22 HIV patients, 10 pulmonary TB patients and 20 controls were stained. Left panel (**a**) shows a representative pseudo-color plot of CD27 versus CD45RA staining among Vδ2 T cells and αβ T cells from one HIV patient. Right panel (**b**) depicts bivariate staining of CD27 and CD45RA among Vδ2 T cell and αβ T cells from one representative healthy control. Notably, as shown in the figure (Fig. 5), thresholds discriminating subsets on the basis of CD27 and CD45RA were defined on αβ T cells and copied to plots expressing these markers on gated Vδ2 T cells

The Vδ2 T cells subsets were further gated and analyzed for the expression of CD27 and CD45RA markers as defined in Fig. 5 to determine the frequency of naïve, memory and effector phenotypes.

**Fig. 6:**
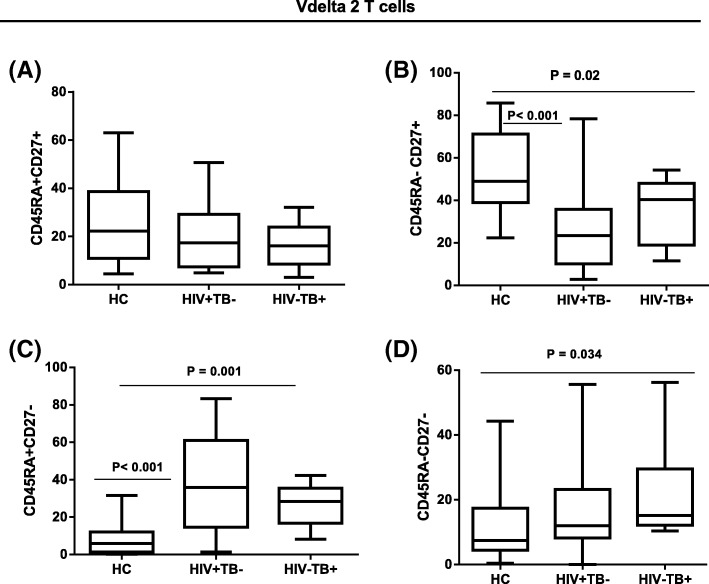
Expression of differentiation markers on Vδ2 subsets. Expression of differentiation markers on Vδ2 T cell naïve (**a**), central memory (**b**), effector cytotoxic (**c**), and effector memory (**d**) subsets from peripheral blood of 22 HIV + TB-, 10 HIV-TB+ patients and 20 healthy controls. *p* values are from the Mann Whitney *U* Test

The median frequency of central memory (CD45RA-CD27+) Vδ2 T cells subsets was significantly higher in healthy controls compared to HIV + TB- (*p* < 0.001) and HIV-TB+ (*p* = 0.02) patients. Though not significant, the frequency of naïve (CD45RA + CD27+) Vδ2 T cells was higher in the healthy controls. In contrast, the median frequency of effector cytotoxic (CD45RA + CD27-) Vδ2 T cells was significantly higher in both patient groups compared to healthy controls (*p* = 0.001). However, we did not find significant differences in the frequency of intermediate effector/effector memory (CD45RA-CD27-) Vδ2 T cells between HIV positive patients and controls. In addition, a higher proportion of these cells was observed in HIV negative pulmonary TB patients (*p* = 0.034) than healthy controls (Fig. 6).

### Functional response of Vδ2 T cells

**Fig. 7:**
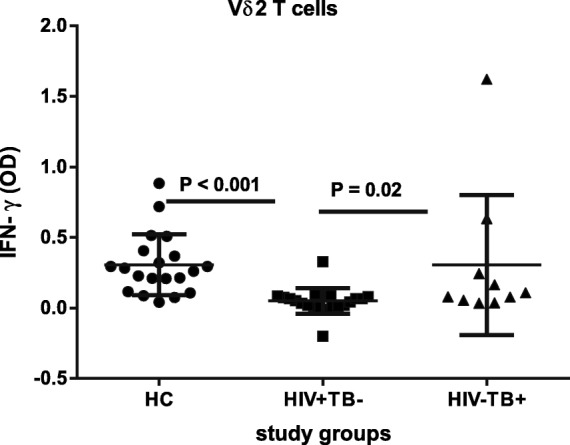
IFN-γ response by Vδ2 T subsets to Isopentenyl Pyrophosphate stimulation. Comparison of median IFN-γ responses by Vδ2 T cells after stimulating with 50 μM IPP for 24 h in 22 HIV + TB-, 10 HIV-TB+ patients and 20 healthy controls. IFN-γ response is depicted as optical density (OD) of the supernatant, as described in materials and methods. *p-*value is from the Mann-Whitney *U* test

The cytokine functional response of Vδ2 T cells was evaluated by measuring the level of IFN-γ production following overnight stimulation with IPP. HIV + TB- patients had the lowest IFN-γ OD level compared to healthy controls and HIV-TB+ patients; no significant difference was observed between HIV-TB+ patients and controls (Fig. 7).

## Discussion

The distribution of total γδ T cells, and Vδ1 and Vδ2 subsets was analyzed in the peripheral blood of HIV and pulmonary TB patients and compared with values from apparently healthy individuals. We also analyzed the expression of activation and adhesion markers on Vδ1 T cells, and exhaustion and differentiation markers and phosphoantigen-specific IFN-γ responses of Vδ2 cells. The median percentage of total γδ T cells was similar between the patients and healthy controls which is in agreement with previous reports [8, 9].

The current study demonstrated an increased proportion of Vδ1 cells in HIV + TB- patients, though of statistically marginally significance compared to controls. Similar results were reported in previous studies, but with greater statistical significance [17, 18]. On the other hand the frequency of Vδ2 cells was significantly lower in HIV + TB- patients compared to healthy controls and HIV-TB+ patients in the current study, such observation was also reported by previous findings [9, 17].

There is no clear consensus with regard to the distribution of Vδ2 T cells in HIV negative pulmonary TB patients and healthy controls. Li et al. [19] showed significantly lower Vδ2 T cells number in pulmonary TB patients compared to controls, Carvalho et al. also reported significantly lower values of these T cells in pulmonary TB patients irrespective of their HIV status [20]. In contrast, there are reports describing increased proportion of Vδ2 T cell subsets in pulmonary TB patients co-infected with HIV [21]. Other studies found no difference in these T cells distribution between pulmonary TB patients and healthy controls [16], similar to our findings in this study. Possibly, the lack of observed consistency in the distribution of Vδ2 T cells in pulmonary TB patients may be related to study dependent differences in the stage of TB disease, or differences in Purified Protein Derivative (PPD) reactivity. Of note, one study showed PPD negative patients had higher counts of γ/δ T cells than those PPD positive patients or controls [15]. Other studies, however, have shown that the distribution of these cells and their subsets was not associated with response to PPD [20]. Other possibly variables contributing to differences between studies include age; some studies enrolled more of adults [15, 20] while other included younger children [16]. The study sample size may contribute as well; we enrolled only limited numbers of pulmonary TB patients, whereas other studies enrolled more subjects [15, 20].

Significantly higher proportions of Vδ1 T cells expressed the activation marker CD38 and adhesion marker CD103 in HIV + TB- patients in this study, consistent with previous reports [7, 9]. This finding is compatible with the involvement of inflammation and stress induced molecules such as MHC class I polypeptide-related sequence A and B (MICA and MICB) known to stimulate these cells and influence their mucosal attachment [2]. In vitro studies demonstrated that HIV can breach the mucosal barrier and facilitate the translocation of viruses and bacteria [22], potentially contributing to local immune reactivity and promoting emigration of these T cell subsets into the peripheral blood, possibly through modulation of some chemokine ligands [23]. Supporting this possibility, a recent study revealed an elevated level of a microbial translocation marker sCD14, which was associated with γδ T cells activation and this activation increased over time [24]. The repertoire analysis of CDR3 length polymorphisms among peripheral and mucosal Vδ1 T cells is an approach which can reveal close lineage relationship of these cells and support the hypothesis of a mucosal activated source of circulating blood Vδ1 cells. Poles et al. study, however, indicated that despite having increased expression of mucosal homing receptors such as CD103 and CCR9 on γδ T cells, the repertoires from peripheral blood and mucosal area appeared to represent two distinct populations in HIV patients [9]. Moreover, in our current study the Vδ1 subset also exhibited significant expression of exhaustion markers CD95 and PD1 in HIV patients suggesting persistent activation of these cells and possibly to altered function. Chronic HIV is associated with reduced effector function of mucosal Vδ1 and Vδ2 T-cells [25].

Despite being asymptomatic and on long term HAART, the current research showed that HIV patients have significantly lower percentages of the Vδ2 subset as well as reduced IFNγ production in response to phosphoantigen (IPP) stimulation, a finding observed in other studies [26, 27]. One possible mechanism for reduced numbers and function of these cells could be direct infection and destruction by HIV-1 itself, at least among those Vδ2 expressing CD4 and chemokine receptors CCR5 and CXCR4 [28] or α4β7 [29]. Of note, increased expression of α4β7 and CCR5 at the surface of activated Vδ2 T cells at close proximity has been found to promote the binding of HIV envelope glycoprotein, which ultimately causes significant killing of these subsets [29]. Additional mechanisms are suggested by our observation of significantly higher expression of CD38, CD95 and PD1 on Vδ2 cells which may reflect an on-going exhaustion or initiation of fas-mediated activation induced cell death. These mechanisms are well described to contribute to loss and/or dysfunction of conventional α/β CD4 and CD8 T cells in HIV patients, and have support in studies of γ/δ T cells [30, 31].

As demonstrated by previous reports [30] and in the current study, the compartmentalization of Vδ2 T cells to subsets of naïve and memory cells according to the expression of markers such as CD45RA and CD27 is not as clear compared with that of conventional α/β T cells. Using gating defined on α/β T cells, we defined Vδ2 T cell subsets as naïve (CD45RA + CD27+), central memory (CD45RA- CD27+), effector memory (CD45RA-CD27-), and effector cytotoxic (CD45RA + CD27-) cells [32]. Previous studies showed HIV patients [30] and pulmonary TB patients [33] had significantly reduced numbers of Vδ2 T cells with the CD45RA-CD27- (effector memory) phenotype. However, in the current and another study [25] there was no significant difference in expression of the effector memory phenotype between HIV patients and healthy controls, and a higher proportion in pulmonary TB patients compared with controls. The overexpression of effector memory cells that we observed in TB patients is particularly relevant in light of observations that Vδ2 cells with the effector memory and effector cytotoxic phenotype expressed relatively abundant IFN-γ [33] and Granzyme B /perforin [32]. Future studies with more detailed functional characterization of Vδ2 subsets in TB patients may therefore shed light into their role as an important component of the host response to TB.

## Conclusion

The current study indicated that the frequency of total γδ T cells was similar among HIV and pulmonary TB patient groups and healthy controls. HIV infection was associated with an increase in the Vδ1 subset and a decrease in the Vδ2 subset frequency and function. Patients with HIV and pulmonary TB infections exhibited significantly higher expression of activation, adhesion and exhaustion markers on Vδ1 T and Vδ2 T cell subsets. HIV negative pulmonary TB patients had significantly higher proportions of effector Vδ2 T cells, consistent with an active role these T cells play in mycobacterial defense and/or pathogenesis. This study is the first of its kind in this country which assessed the distribution of these T cells. However, further longitudinal studies evaluating phenotypic and functional properties of γδ T cells in patients before and after therapy, as well as the inclusion of co-infected patients and larger sample size may help to resolve underlying etiologies contributing to altered distributions of γδ T lymphocytes and their role in the pathogenesis of the diseases in these patients.

## Abbreviations

AAU: Addis Ababa University
AFB: Acid fast bacilli
AHRI: Armauer Hanson research institute
ALERT: All African leprosy and TB rehabilitation and training center
BD: Becton Dickinson
CCR: Chemokine receptor
CD: Cluster of differentiation
DC: Dendritic cell
ELISA: Enzyme linked immunosorbent assay
FACS: Florescence activated cell sorter
HAART: Highly active antiretroviral therapy
HIV: Human immunodeficiency virus
HLA: Human leukocyte antigen
IFN-γ: Interferon gamma
IPP: Isopentenyl pyrophosphate
MHC: Major histocompatibility complex
MTB: Mycobacterium tuberculosis
N-CAM: Neural cell adhesion molecule
OD: Optical density
PBS: Phosphate buffer saline
PD1: Program death
PPD: Purified protein derivative
SPSS: Statistical Package for Social Sciences
TCR: T cell receptor
Th: T helper cell
VCT: Voluntary counseling and testing
γδ T: Gamma delta T cell

## References

[1] Carding SR, Egan PJ (2002). [Gamma][delta] T cells: functional plasticity and heterogeneity. Nat Rev Immunol.

[2] Groh V, Steinle A, Bauer S, Spies T (1998). Recognition of stress-induced MHC molecules by intestinal epithelial γδ T cells. Science.

[3] Chen ZW, Letvin NL (2003). Vγ2Vδ2+ T cells and anti-microbial immune responses. Microbes Infect.

[4] Dieli F, Sireci G, Caccamo N, Di Sano C, Titone L, Romano A (2002). Selective depression of interferon-γ and Granulysin production with increase of proliferative response by Vγ9/Vδ2 T cells in children with tuberculosis. J Infect Dis.

[5] Poccia F, Battistini L, Cipriani B, Mancino G, Martini F, Gougeon ML (1999). Phosphoantigen-reactive Vγ9Vδ2 T lymphocytes suppress in vitro human immunodeficiency virus type 1 replication by cell-released antiviral factors including CC chemokines. J Infect Dis.

[6] Poccia F, Agrati C, Montesano C, Martini F, Pauza C, Fisch P (2002). Innate T-cell immunity in HIV infections: the role of Vg9Vd2 T lymphocytes. Curr Mol Medic.

[7] Le Roy GMFA, JP FM, JP CEHJF, Helluin O, Fukushima N, Bouchart FZC (1996). Similarity of expression of activation markers and CD28 on gamma delta and alpha beta-receptor T cells in HIV infection. Clin Immunol Immunopathol.

[8] Hinz T, Wesch D, Friese K, Reckziegel A, Arden B, Kabelitz D (1994). T cell receptor γδ repertoire in HIV-1-infected individuals. Eur J Immunol.

[9] Poles MA, Barsoum S, Yu W, Yu J, Sun P, Daly J (2003). Human immunodeficiency virus type 1 induces persistent changes in mucosal and blood γδ T cells despite suppressive therapy. J Virol.

[10] Li H, Peng H, Ma P, Ruan Y, Su B, Ding X (2008). Association between Vγ2Vδ2 T cells and disease progression after infection with closely related strains of HIV in China. Clin Infect Dis.

[11] Agrati C, D’Offizi G, Gougeon ML, Malkovsky M, Sacchi A, Casetti R (2011). Innate gamma/Delta T-cells during HIV infection: Terra relatively incognita in novel vaccination strategies?. AIDS Rev.

[12] Boullier S, Cochet M, Poccia F, Gougeon ML (1995). CDR3-independent gamma delta V delta 1+ T cell expansion in the peripheral blood of HIV-infected persons. J Immunol.

[13] Ito M, Kojiro N, Ikeda T, Ito T, Funada J, Kokubu T (1992). Increased proportions of peripheral blood γδ T cells in patients with pulmonary tuberculosis. Chest.

[14] Rojas RE, Chervenak KA, Thomas J, Morrow J, Nshuti L, Zalwango S (2005). Vδ2+ γδ T cell function in Mycobacterium tuberculosis– and HIV-1–positive patients in the United States and Uganda: application of a whole-blood assay. J Infect Dis.

[15] Baliko Z, Szereday L, Szekeres-Bartho J (1997). Gamma/delta T lymphocytes in Mycobacterium tuberculosis infection. Thorax.

[16] Dieli F, Sireci G, Di Sano C, Romano A, Titone L, Di Carlo P (2000). Ligand-specific αβ and γδ T cell responses in childhood tuberculosis. J Infect Dis.

[17] Wesch D, Hinz T, Kabelitz D (1998). Analysis of the TCR Vgamma repertoire in healthy donors and HIV-1-infected individuals. Int Immunol.

[18] Rossol R, Dobmeyer JM, Dobmeyer TS, Klein SA, Rossol S, Wesch D (1998). Increase in Vδ1+ γδ T cells in the peripheral blood and bone marrow as a selective feature of HIV-1 but not other virus infections. Br J Haematol.

[19] Li B, Rossman MD, Imir T, Oner-Eyuboglu AF, Lee CW, Biancaniello R (1996). Disease-specific changes in gammadelta T cell repertoire and function in patients with pulmonary tuberculosis. J Immunol.

[20] Carvalho A, Matteelli A, Airo P, Tedoldi S, Casalini C, Imberti L (2002). γδ T lymphocytes in the peripheral blood of patients with tuberculosis with and without HIV co-infection. Thorax.

[21] Ruiz P, Geraldino N (1995). Peripheral γδ T-cell population in HIV-infected individuals with mycobacterial infection. Cytometry.

[22] Nazli A, Chan O, Dobson-Belaire WN, Ouellet M, Tremblay MJ, Gray-Owen SD (2010). Exposure to HIV-1 directly impairs mucosal epithelial barrier integrity allowing microbial translocation. PLoS Pathog.

[23] Poggi A, Carosio R, Fenoglio D, Brenci S, Murdaca G, Setti M (2004). Migration of Vδ1 and Vδ2 T cells in response to CXCR3 and CXCR4 ligands in healthy donors and HIV-1–infected patients: competition by HIV-1 tat. Blood.

[24] Li Z, Li W, Li N, Jiao Y, Chen D, Cui L (2014). γδ T cells are involved in acute HIV infection and associated with AIDS progression. PLoS One.

[25] Cimini E, Agrati C, D’Offizi G, Vlassi C, Casetti R, Sacchi A (2015). Primary and chronic HIV infection differently modulates mucosal Vδ1 and Vδ2 T-cells differentiation profile and effector functions. PLoS One.

[26] Bordon J, Evans PS, Propp N, Davis CE, Redfield RR, Pauza CD (2004). Association between longer duration of HIV-suppressive therapy and partial recovery of the Vγ2 T cell receptor repertoire. J Infect Dis.

[27] Martini F, Poccia F, Goletti D, Carrara S, Vincenti D, D’Offizi G (2002). Acute human immunodeficiency virus replication causes a rapid and persistent impairment of Vγ9Vδ2 T cells in chronically infected patients undergoing structured treatment interruption. J Infect Dis.

[28] Imlach S, Leen C, Bell JE, Simmonds P (2003). Phenotypic analysis of peripheral blood γδ T lymphocytes and their targeting by human immunodeficiency virus type 1 in vivo. Virology.

[29] Li H, Chaudry S, Poonia B, Shao Y, Pauza CD (2013). Depletion and dysfunction of Vγ2Vδ2 T cells in HIV disease: mechanisms, impacts and therapeutic implications. Cell Mol Immunol.

[30] Cummings J-S, Cairo C, Armstrong C, Davis CE, Pauza CD (2008). Impacts of HIV infection on Vγ2Vδ2 T cell phenotype and function: a mechanism for reduced tumor immunity in AIDS. J Leukoc Biol.

[31] Yang Y, Dong B, Mittelstadt PR, Xiao H, Ashwell JD (2002). HIV tat binds Egr proteins and enhances Egr-dependent transactivation of the Fas ligand promoter. J Biol Chem.

[32] Angelini DF, Borsellino G, Poupot M, Diamantini A, Poupot R, Bernardi G (2004). FcγRIII discriminates between 2 subsets of Vγ9Vδ2 effector cells with different responses and activation pathways. Blood.

[33] Gioia C, Agrati C, Casetti R, Cairo C, Borsellino G, Battistini L (2002). Lack of CD27− CD45RA− Vγ9Vδ2+ T cell effectors in immunocompromised hosts and during active pulmonary tuberculosis. J Immunol.

